# Adherence to option B + antiretroviral therapy and associated factors in pregnant and breastfeeding women in Sub-Saharan Africa: a systematic review and meta-analysis

**DOI:** 10.1186/s12889-023-17004-9

**Published:** 2024-01-05

**Authors:** Lucresse Corine Fassinou, Diane Songwa Nkeunang, Thérèse Delvaux, Nicolas Nagot, Fati Kirakoya-Samadoulougou

**Affiliations:** 1 INSSA, Université Nazi Boni, Bobo-Dioulasso, Burkina Faso; 2 Centre de Recherche en Epidémiologie, Biostatistique Et Recherche Clinique, Ecole de Santé Publique, Université Libre de Bruxelles, Brussels, Belgique; 3 Institute of Tropical Medicine, Department of Public Health, Antwerp, Belgium; 4 Pathogenesis & Control of Chronic and Emerging Infections, Univ. Montpellier, INSERM, Univ. Antilles, Etablissement Français du Sang, Montpellier, France

**Keywords:** Adherence, Antiretroviral therapy, Pregnancy, Breastfeeding, Sub-Saharan Africa, Option B +

## Abstract

**Background:**

To assess the adherence to option B + antiretroviral therapy (ART) and associated factors in pregnant and breastfeeding women in Sub-Saharan Africa (SSA).

**Methods:**

We conducted a comprehensive search from 01^st^ January 2012 to 03^rd^ October 2022, across four databases: PubMed, Scopus, Proquest Central, and Index Medicus Africain, to identify studies focused on pregnant and/or breastfeeding women living with HIV and receiving option B+ ART in SSA. Studies reporting adherence data were included in the meta-analysis. Were excluded studies published before 01^st^ January 2012, grey literature, systematic reviews, and meta-analysis studies. Articles selection and data extraction were performed independently by two reviewers. We evaluated pooled adherence and pooled association between various factors and adherence using a random-effects model.

**Results:**

Overall, 42 studies involving 15,158 participants across 15 countries contributed to the meta-analysis. The overall pooled adherence was 72.3% (95% CI: 68.2–76.1%). Having high education level (pooled odds ratio (OR): 2.25; 95% CI: 1.57–3.21), living in urban area (pooled OR: 1.75; 95% CI: 1.10–2.81), disclosing status to a family/partner (pooled OR: 1.74; 95% CI: 1.27–2.40), having a support system (pooled OR: 3.19; 95% CI: 1.89–5.36), receiving counseling (pooled OR: 3.97; 95% CI: 2.96–5.34), initiating ART at early clinical HIV stage (pooled OR: 2.22; 95% CI: 1.08–4.56), and having good knowledge on PMTCT/HIV (pooled OR: 2.71; 95% CI: 1.40–5.25) were factors significantly associated with adherence to option B + ART.

**Conclusions:**

Despite the implementation of option B+ ART, the level of adherence among pregnant and breastfeeding women in SSA falls short of meeting the critical thresholds for viral load suppression as outlined in the 95-95-95 objectives set for 2025. These objectives are integral for achieving HIV elimination, and in turn, preventing HIV mother-to-child transmission. To bridge this gap, urgent tailored interventions based on individual and structural factors are essential to enhance adherence within these subgroups of women. This targeted approach is crucial in striving towards the HIV elimination target in SSA.

**Supplementary Information:**

The online version contains supplementary material available at 10.1186/s12889-023-17004-9.

## Background

Approximately, 1.3 million HIV-positive women worldwide become pregnant each year [1]. Of these, around 91% reside in Sub-Saharan Africa (SSA) with highest HIV burden in women [2, 3]. Globally, remarkable progress has been seen over the years in reducing new HIV infection among children under five years, from 320,000 in 2010 to 160,000 in 2021 [4]. However, further reduction is needed based on the global commitments to eliminate HIV by 2030, with a focus on mother to-child transmission (MTCT) [5]. According to the World Health Organization (WHO), HIV vertical transmission elimination in a country is defined based on the reduction of MTCT rate below 2% and 5% in non-breastfeeding and breastfeeding populations respectively, and the reduction of new paediatric HIV infections due to MTCT to less than 50 cases per 100,000 live births [6].

The observed decline in new infection in children under five years of age could be largely attributed to the different antiretroviral therapy (ART) strategies recommended by the WHO as part of the prevention of mother-to-child transmission (PMTCT) program. The most recent strategy includes the option B + strategy, initially conceived and implemented in Malawi in 2011, and rapidly adopted since 2013 by many SSA countries [7–9]. Option B + recommends lifelong ART for pregnant or breastfeeding women as soon as they test positive, regardless of gestational age, WHO clinical stage, and CD4 cell count [10, 11], and appears to be an effective PMTCT strategy as it has a significant impact on treatment uptake and improves outcomes for mothers and exposed infants [12]. In SSA, the ART coverage in pregnant women increased from 17% in 2010 to 87% in 2020, with HIV vertical transmission decreasing by 56% over the same period [13]. However, despite the success since the implementation of the option B + strategy, adherence to the regimen remains the main challenge that many countries still face, compromising the effectiveness of the strategy. The periods of pregnancy and breastfeeding represent a window of time associated with an increase in poor adherence, which leads to increased risks such as viral suppression failure, HIV progression, and the development of drug resistance, with these consequences heightening the risk of MTCT [14, 15].

Many studies in SSA have focused on the magnitude of adherence to option B + ART and the factors associated with this adherence. The results of these studies revealed disparities in the prevalence of ART adherence across different countries. Age [16–18], educational level [14, 19], experience of side effects [20, 21], knowledge of PMTCT or HIV [20], support from partner [16, 20, 21], disclosure to a family member or partner [11, 14, 22–24], receiving counseling [25], no fear of stigma [21], and employment status [26] were the factors reported to be associated with ART adherence.

In 2019, the UNICEF report revealed a vertical HIV transmission rate of 12.2% in SSA, despite reasonably good ART coverage in PMTCT programs [27]. Achieving optimal adherence to ART is essential to suppress the virus and prevent vertical transmission [28]. In the context of the global movement involving the use of option B + triple ART prophylaxis during pregnancy and breastfeeding for the prevention of HIV vertical transmission [29], a critical research question emerges: What is the extent of adherence to option B + ART prophylaxis among pregnant and breastfeeding women in Sub-Saharan Africa (SSA), and what factors contribute to or hinder optimal adherence in this specific population? Regarding the lack of comprehensive and synthetized evidence on this problematic, our systematic review and meta-analysis study, aimed at filling this knowledge gap and provide evidence-based perspectives, essential for designing targeted interventions to improve ART adherence. This research could serve as a compass to help advance the overall goal of curbing vertical HIV transmission and improving the well-being of women living with HIV in SSA.

## Methods

This systematic review and meta-analysis was conducted using the Preferred Reporting Item for Systematic Review and Meta-analysis (PRISMA) guidelines [30]. To avoid duplication, we registered the protocol in PROSPERO with the registration number CRD42022346122 on 24 July 2022.

### Information sources and search strategy

Four (04) electronic databases: PubMed, Scopus, Proquest Central, and Index Medicus Africain (IMA), were used in the study. The queries were developed using five (05) main keywords: a) adherence, b) prevention of mother-to-child transmission, c) antiretroviral therapy or option B, d) pregnant or breastfeeding women, and e) countries of sub-Saharan Africa (see the full search strategy and the results of the search for each database in the supplementary material). Two authors (LCF and DS) conducted searches of these databases. Terms were searched separately and together using Boolean operator “OR” or “AND”. Articles were filtered using the publication date (articles published from 01^st^ January 2012 to 03^rd^ October 2022). The date of 01^st^ January 2012 was chosen because option B + strategy was first implemented in the late 2011 in SSA (Malawi) [31]. The reference manager software, Endnote X9.3.3 (bld 13966), was used to export different articles obtained from the searches and remove duplicates.

### Eligibility criteria

All types of studies (observational, clinical trial) were included in the analysis if they met all the following criteria: i) the study included pregnant and/or breastfeeding women living with HIV in one or more SSA countries; ii) participants were administered option B + ART; and iii) the adherence rate to the treatment was clearly quantified. All studies that considered women in general and that did not give information on the sub-group of pregnant and/or breastfeeding women were excluded from the meta-analysis. Articles published before 01^st^ January 2012 were excluded to avoid studies in the pre-option B + era. Moreover, grey literature (thesis, dissertations, conference papers, books, and reports) as well as systematic reviews and meta-analyses were not considered in this meta-analysis.

### Study selection

All abstracts and full-text articles were independently reviewed by two authors (LCF and DS) according to the eligibility criteria using rayyan web-tool for systematic reviews [32]. Discrepancies were resolved by reaching an agreement by consensus.

### Data extraction

LCF and DS independently extracted and compared data through an extraction form developed using kobo toolbox software [33]. For each paper, details were extracted on publication details (first author’s name, publication year), study characteristics (data collection years, country where the study was performed, design of the study, sample size), participant characteristics (age, type of women included in the study: pregnant women only, breastfeeding women only, or both), adherence measure characteristics (method of measure, threshold used for defining adherence, number of adherent participants, time frame used to measure adherence), and factors associated with adherence (a total of 10 relevant factors were considered in this study: age, educational level, occupation, area of residence, marital status, support of anyone or involvement from a partner, disclosure status, receiving counseling, stage of HIV/AIDS, and knowledge on PMTCT/HIV).

### Study outcomes

The primary outcome of this study was the level of adherence to option B + ART. Adherence was estimated in each study by dividing the number of individuals with good adherence by the total number of individuals included in the study. The overall pooled adherence was determined based on the definition and adherence thresholds adopted in each study. A minimum threshold of 80% was considered. When a study used more than one threshold, only values related to a threshold of 80% were considered. When more than one measurement method was used in a study, only data concerning the most objective method were considered for analysis (for example: Dried blood spots > pill count > pharmacy refill > self-reported adherence).

The second outcome, which was related to the identification of factors associated with adherence, was determined using the odds ratio (OR) derived from binary outcomes. The overall pooled OR was then calculated. Studies that did not provide the necessary data for OR calculation were excluded from the combined analysis (calculation of pooled OR).

### Quality assessment of the studies

The quality of the studies included in the meta-analysis was assessed using the Newcastle–Ottawa Scale (NOS) quality assessment form for observational studies recommended by the Agency for Healthcare Research and Quality (AHRQ) [34]. This scale uses a star system to assess the quality of a study in three domains: the selection of study participants (maximum 5 stars), comparability (maximum 1 star), and ascertainment of outcomes (maximum 3 stars). This form had the following criteria: representativeness, sample size justification, non-response, ascertainment of exposure, control for confounding, ascertainment of outcome, and statistical tests. The total score ranges from 0 to 9 stars. The quality of studies were indicated based on the number of stars (9 stars: very good; 7–8 stars: good; 5–6 stars: satisfactory; 0–4 stars: unsatisfactory) [34].

### Statistical methods and analysis

The extracted data were analysed using R version 4.2.0. Pooled adherence to option B + ART was estimated using DerSimonian and Laird’s method with a random-effects model [35]. Heterogeneity between studies was assessed using Cochran’s *Q* test, and its magnitude was evaluated using Higgins *I* ^*2*^ statistics [36]. Publication bias was evaluated using funnel plots and Egger’s test [37].

To investigate potential sources of heterogeneity, we performed subgroup analyses and stratified our data according to the women’s status categories considered in the study (pregnancy, breastfeeding, pregnancy and breastfeeding), the year of publication (before 2018, after 2018), the SSA region (eastern, southern, western, central), the adherence recall time frame (last 7 days, last 15–30 days, last 90 days), study design (cross-sectional, cohort, randomised control trial [RCT]), and the instrument of measure used (self-report, pill count/pharmacy refill, combined measure, and others). A combined measure was defined as more than two (02) measures used simultaneously in a study. Concerning the subgroup analysis based on the instrument measure, one study [38] did not report the information associated with the instrument used and was not considered for this particular analysis. The stratification of the years of publication (before 2018 and after 2018) was based on the purpose to compare recent publications (articles published in the last five years) to less recent publications (articles published before the last five years).

Further, the potential source of heterogeneity was investigated using a random-effects meta-regression analysis to assess the associations between adherence from every studies and the study characteristics: women’s status, year of publication, instrument of measure, time frame, region, and the quality of the study.

## Results

### Study selection

**Fig. 1:**
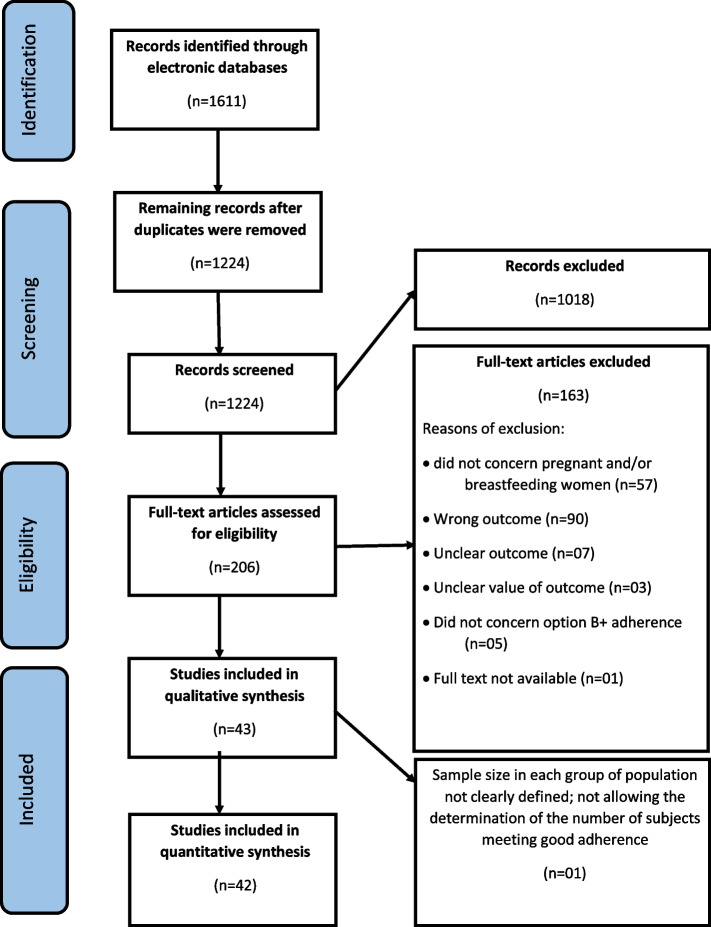
PRISMA flowchart of studies included in the systematic review and meta-analysis of adherence to option B + ART among pregnant and/or breastfeeding women in SSA

After searching different electronic databases, we found 1611 records, of which 206 were deemed relevant for full-text eligibility assessment. Finally, 42 studies were included in the quantitative analysis (Fig. 1) [11, 14, 16–26, 38–66].

### Study characteristics

A total of 15,595 participants with available outcome data from the 43 studies were included in the qualitative analysis. These studies were conducted between 2013 and 2020 and published between 2014 and 2022. Most of the studies (17 out of 43) were conducted in Eastern African countries, followed by Austral African countries (14 out of 43), and West African countries (08 out of 43). One study was conducted in two countries (South Africa and Uganda) [67] and the other in three countries (Mozambique, South Africa, and Mali) [46]. The sample size varied between 42 [18] and 1592 [22] and more than 50% (22 out of 43) of the studies involved only pregnant women.

Out of the 43 eligible studies, 42 (97.7%) clearly reported instrument of adherence measures. Composite measure (combination of at least two different measures to produce one measure) was used in 7 studies [19, 22, 25, 26, 42, 47, 62]. Thirty-four studies [11, 14, 16–18, 20, 21, 23, 24, 40, 41, 43–46, 48–61, 63–67] used only one measure, while one study used three separate measures [Dried Blood Spot (DBS); self-report questionnaire and self-report using Visual Adherence Scale (VAS)] [39]. For this study, only DBS values were considered in the analysis because the DBS measure is more objective and precise than SR measures [39]. Globally, more than half (32 out of 43 studies) of the included studies used self-reporting (SR) as a measure of adherence, either alone (26 studies) or in combination (06 studies). The instruments used to measure self-reported adherence were as follows: simple questionnaire in most of the studies (19 studies), Visual Adherence Scale in two studies [24, 44], Center for Adherence Support Evaluation (CASE) in two studies [40, 65] and Adult AIDS Clinical Trial Group (AACTG) in three studies [52, 57, 58]. The composite measures used to measure adherence were: pharmacy refill and self-report in one study [22], pill count and self-report in four studies [25, 26, 47, 62], and a combination of self-reported questionnaire, pharmacy refill, and Visual Adherence Scale in one study [42].

Different thresholds were used to define adherence across the studies, ranging from 80–100%. Two studies used the CASE scale defined adherence based on the score threshold (> 10 [40]and > 11 [65]) instead of the percentage threshold. Moreover, a study reported adherence based on biological measurements (DBS) and did not use a threshold.

Adherence recall timeframes ranged from as recent as “the last day” [17] to as far as “the last 90 days” [22, 48, 49]. Six studies lacked clear descriptions of the timeframe used [21, 46, 47, 50, 51, 65]. The study designs were: cross-sectional (29 studies), cohort (9 studies), and controlled clinical trial (5 studies).

Eleven studies did not identify the factors associated with option B + ART adherence [24, 39, 42, 46, 48, 49, 54–56, 59, 67]. Table 1 presents the characteristics of the studies included in the systematic review and meta-analysis in further detail.

**Table 1:** Characteristics of studies included in the systematic review and meta-analysis of adherence to option B + ART in SSA

N°	Author	Country	Study design	Year of publication	Year of data collection	Study population (women)	N in analysis	Instrument of adherence measure	Recall time frame (in days)	Study evaluated associated factors
1	Abdisa et al. [16]	Ethiopia	Cross-sectional	2021	2019	Pregnant and breastfeeding	254	Self -Report	03	Yes
2	Abebe et al. [40]	Ethiopia	Cross-sectional	2022	2018	pregnant	368	Self-Report (Center for Adherence Support Evaluation (CASE) index)	07	Yes
3	Adeniyi et al. [22]	South Africa	Cross-sectional	2018	2016	pregnant	1592	Pharmacy refill + Self-Report	30	Yes
4	Adeniyi et al. [17]	South Africa	Cross-sectional	2020	2018	Breastfeeding	485	Self –Report	01	Yes
5	Aduloju et al. [19]	Nigeria	Cross-sectional	2020	2018–2020	Pregnant	170	Pill count + modified Morisky Medication Adherence Scale (MMAS)	30	Yes
6	Aferu et al. [41]	Ethiopia	Cross-sectional	2020	2018	Pregnant	103	Self-Report	90	Yes
7	Agboeze et al. [20]	Nigeria	Cross-sectional	2018	2016	Pregnant	268	Self –Report	03	Yes
8	Alcaide et al. [39]	South Africa	Controlled clinical trial	2017	Not reported	Pregnant	379	Dry Blood Spot (DBS)	03	No
9	Aregbesola et al. [65]	Nigeria	Cross-sectional	2018	2015	Pregnant	126	Self –Report (CASE)	Not reported	Yes
10	Asefa et al. [23]	Ethiopia	Cross-sectional	2020	2018	Pregnant and breastfeeding	180	Self -Report	03	Yes
11	Atanga et al. [42]	Cameroon	Cohort	2018	2013–2015	Pregnant and breastfeeding	185	Self –Report (questionnaire) + pharmacy refill + Self –Report (Visual Analogue Scale (VAS) tool)	30	No
12	Brittain et al. [43]	South Africa	Cross-sectional	2018	2013–2017	Pregnant	482	Self –Report	30	Yes
13	Calder et al. [44]	Nigeria	Controlled clinical trial	2020	2013–2015	Pregnant	210	Self –Report (VAS tool)	15	Yes
14	Dada et al. [14]	Nigeria	Cross-sectional	2021	Not reported	Pregnant and breastfeeding	284	Self –Report + pill count	30	Yes
15	Decker et al. [18]	Uganda	Cohort	2017	2013	Breastfeeding	42	Self –Report + pill count	30	Yes
16	Ebuy et al. [11]	Ethiopia	Cross-sectional	2014	2014	Pregnant	263	Self –Report	03	Yes
17	Erlwanger et al. [45]	Zimbabwe	Controlled clinical trial	2017	2014–2015	Pregnant and breastfeeding	1113	Medication Possession Ratio (MPR)	30	Yes
18	Fedlu et al. [25]	Ethiopia	Cross-sectional	2020	2019	Pregnant and breastfeeding	190	Self –Report + pill count	30	Yes
19	Fernandez-Luiz et al. [46]	Mozambique; south Africa; Mali	Cohort	2022	2018–2020	Pregnant	99	Self –Report	Not reported	No
20	Gebretsadik et al. [47]	Ethiopia	Cross-sectional	2020	2017	Pregnant and breastfeeding	350	Self –Report	Not reported	Yes
21	Haas et al. [48]	Malawi	Cohort	2016	2011–2013	Pregnant and breastfeeding	765	Electronic Medical Records System	90	No
22	Itoua et al. [38]	Republic of Congo	Cross-sectional	2015	2014	Pregnant and breastfeeding	130	Not reported	07	Yes
23	Kadima et al. [26]	Lesotho	Cohort	2018	2016	Pregnant	107	pill count	03	Yes
24	Larsen et al. [49]	South Africa	Cohort	2019	2012–2014	breastfeeding	1572	Self –Report	90	No
25	Matthews et al. [67]	South Africa; Uganda	Cohort	2020	2015–2017	Pregnant and breastfeeding	437	Real-time Electronic Adherence Monitor	01	No
26	Mukose et al. [24]	Uganda	Cohort	2021	2013–2016	Pregnant and breastfeeding	410	Self –Report (VAS)	30	No
27	Mukosha et al. [50]	Zambia	Cross-sectional	2020	Not reported	Pregnant	71	MPR	Not reported	Yes
28	Nsubuga-Nyombi et al. [51]	Uganda	Cross-sectional	2018	Not reported	Pregnant and breastfeeding	122	Self –Report	Not reported	Yes
29	Nutor et al. [66]	Zambia	Cross-sectional	2020	2016	Pregnant and breastfeeding	150	Self-Report	07	Yes
30	Omonaiye et al. [52]	Nigeria	Cross-sectional	2019	2018	Pregnant	275	Self –Report (Adult AIDS Clinical Trial Groups (AACTG) standardized survey)	04	Yes
31	Omonaiye et al. [53]	Nigeria	Cross-sectional	2019	2018	Pregnant	275	Pharmacy refill	02	
32	Onono et al. [54]	Kenya	Cross-sectional	2020	2017	breastfeeding	200	Self –Report	30	No
33	Phillips et al. [55]	South Africa	Cross-sectional	2017	2013	Pregnant and breastfeeding	452	Self –Report	30	Yes
34	Phillips et al. [56]	South Africa	Controlled clinical trial	2016	2013–2014	Pregnant and breastfeeding	517	Self –Report	30	No
35	Ramlagan et al. [57]	South Africa	Cross-sectional	2018	2014–2015	Pregnant	673	Self –Report (AACTG)	04	No
36	Ramlagan et al. [58]	South Africa	Controlled clinical trial	2019	2014–2017	Pregnant	683	Self –Report (AACTG)	04	Yes
37	Schnack et al. [59]	Uganda	Cohort	2016	Not reported	Pregnant	76	Pill count	30	No
38	Tarekegn et al. [60]	Ethiopia	Cross-sectional	2019	2017	Pregnant	293	Self –Report	03	Yes
39	Tesfaye et al. [61]	Ethiopia	Cross-sectional	2019	2017	Pregnant	290	Self –Report	03	Yes
40	Tsegaye et al. [62]	Ethiopia	Cross-sectional	2016	2016	Pregnant and breastfeeding	190	Self –Report + pill count	30	Yes
41	Wondimu et al [21]	Ethiopia	Cross-sectional	2020	2018	Pregnant	347	Self –Report	Not reported	Yes
42	Zacharius et al [63]	Tanzania	Cross-sectional	2019	2017	Pregnant and breastfeeding	305	Pill count	30	Yes
43	Zoungrana-Yameogo et al. [64]	Burkina-Faso	Cross-sectional	2022	2019-2020	Pregnant and breastfeeding	112	Self -Report	30	Yes

### Quality assessment of studies

The quality assessment was limited to studies included in the quantitative analysis. Of these studies (42 in total), over half (28 studies) demonstrated satisfactory to very good quality (Table 2). When considering the selection bias domain, 13 studies showed a high risk of bias regarding sample representativeness, 24 studies in terms of sample size justification, 29 studies in terms of non-response, and 15 studies in terms of adherence measure tool ascertainment. Under the comparability domain, 7 studies exhibited high risk of bias regarding control of confounding factors. In the outcome domain, none of the studies demonstrated high risk of bias regarding outcome ascertainment, while 10 studies showed high risk of bias in reporting statistical test.

**Table 2:** Quality assessment of the studies included in the quantitative analysis (*N* = 42)

N°	Author	Representativeness	Sample size justification	Non-response	Ascertainment of exposure	Control for confounding	Assessment of outcomes	Statistical tests	Overall quality
1	Abdisa et al. [16]	0 star	*	*	*	*	*	*	satisfactory
2	Abebe et al. [40]	*	*	0 star	**	*	*	*	satisfactory
3	Adeniyi et al. [22]	*	0 star	0 star	**	*	**	*	satisfactory
4	Adeniyi et al. [17]	*	*	*	**	*	*	*	good
5	Aduloju et al. [19]	*	*	*	**	*	**	*	good
6	Aferu et al. [41]	*	*	0 star	0 star	0 star	*	0 star	unsatisfactory
7	Agboeze et al. [20]	*	*	*	*	0 star	*	0 star	satisfactory
8	Alcaide et al. [39]	*	0 star	0 star	**	*	**	*	satisfactory
9	Aregbesola et al. [65]	0 star	*	*	**	*	*	*	satisfactory
10	Asefa et al. [23]	*	*	*	*	*	*	*	satisfactory
11	Atanga et al. [42]	0 star	0 star	0 star	*	*	*	*	unsatisfactory
12	Brittain et al. [43]	0 star	0 star	0 star	0 star	*	*	0 star	unsatisfactory
13	Calder et al. [44]	*	*	0 star	**	*	*	*	satisfactory
14	Dada et al. [14]	*	0 star	0 star	0 star	*	*	*	unsatisfactory
15	Decker et al. [18]	*	0 star	0 star	0 star	0 star	*	*	unsatisfactory
16	Ebuy et al. [11]	*	*	*	*	*	*	*	satisfactory
17	Erlwanger et al. [45]	0 star	0 star	0 star	**	*	**	0 star	unsatisfactory
18	Fedlu et al. [25]	*	*	*	0 star	*	*	*	satisfactory
19	Fernandez-Luiz et al. [46]	*	0 star	0 star	0 star	Non applicable	*	0 star	unsatisfactory
20	Gebretsadik et al. [47]	*	*	*	0 star	*	*	*	Satisfactory
21	Haas et al. [48]	*	0 star	0 star	**	*	**	*	satisfactory
22	Itoua et al. [38]	*	0 star	0 star	0 star	0 star	*	0 star	unsatisfactory
23	Kadima et al. [26]	0 star	0 star	0 star	0 star	*	*	0 star	unsatisfactory
24	Larsen et al. [49]	*	*	0 star	0 star	*	*	*	satisfactory
25	Mukose et al. [24]	*	0 star	0 star	**	*	*	*	satisfactory
26	Mukosha et al. [50]	0 star	0 star	0 star	**	*	**	*	satisfactory
27	Nsubuga-Nyombi et al. [51]	*	0 star	0 star	0 star	*	*	0 star	unsatisfactory
28	Nutor et al	0 star	0 star	0 star	*	*	*	*	unsatisfactory
29	Omonaiye et al. [52]	*	*	0 star	0 star	*	*	*	satisfactory
30	Omonaiye et al. [53]	*	*	0 star	**	*	*	*	satisfactory
31	Onono et al. [54]	0 star	0 star	0 star	*	*	*	*	unsatisfactory
32	Phillips et al. [55]	0 star	0 star	0 star	*	*	*	*	unsatisfactory
33	Phillips et al. [56]	0 star	0 star	0 star	**	0 star	*	0 star	unsatisfactory
34	Ramlagan et al. [57]	*	0 star	0 star	**	*	*	*	satisfactory
35	Ramlagan et al. [58]	*	0 star	0 star	**	*	*	0 star	satisfactory
36	Schnack et al. [59]	0 star	0 star	0 star	*	0 star	**	*	unsatisfactory
37	Tarekegn et al. [60]	*	*	*	0 star	*	*	*	satisfactory
38	Tesfaye et al. [61]	*	*	*	*	*	*	*	satisfactory
39	Tsegaye et al. [62]	*	*	*	0 star	*	*	*	satisfactory
40	Wondimu et al [21]	0 star	*	*	0 star	*	*	*	satisfactory
41	Zacharius et al [63]	*	0 star	0 star	**	*	*	*	satisfactory
42	Zoungrana-Yameogo et al. [64]	*	0 star	0 star	*	*	*	*	satisfactory

### Overall adherence to option B + ART

**Fig. 2:**
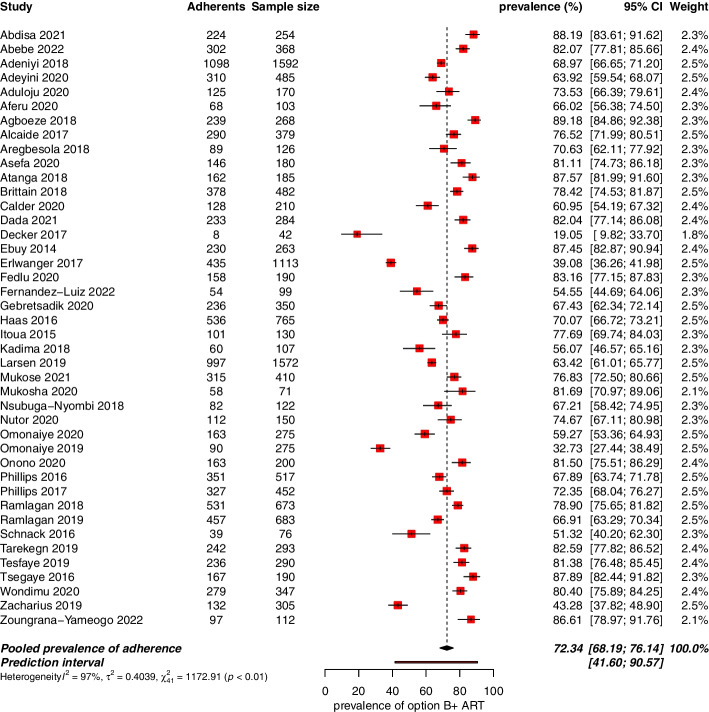
Pooled proportion of pregnant and/or breastfeeding women adhering to option B + ART in SSA

**Fig. 3:**
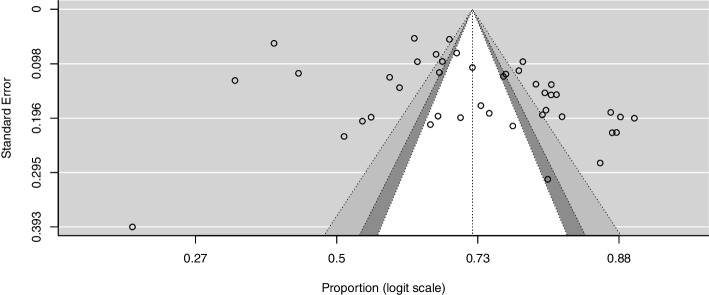
Funnel plot of the pooled adherence to option B + ART in pregnant and/or breastfeeding women in SSA

Overall, 42 studies (involving 15,158 participants across 15 countries) were included in the quantitative analysis. Proportions of adherence to option B + ART and 95% CIs from individual studies with the pooled estimate are presented in the Fig. 2. These proportions ranged from 19% in western Uganda [18] to 89.2% in Southern Ethiopia [20]. The overall pooled adherence estimate was 72.3% (95% CI: 68.2–76.1%; *I* ^*2*^ = 96.5%; *p* < 0.01) (Fig. 2). The funnel plot revealed an asymmetric repartition of the studies (Fig. 3), although the Egger’s test did not yield statistical significance (*z* = 0.87; *p* = 0.38).

### Subgroup analysis of adherence to option B + ART

Table 3 outlines the detailed results of the subgroup analysis. Stratified by women’s status, the overall pooled adherence prevalence was 71.9% (95% CI: 66.8–76.4%) for pregnant women and 61.1% (95% CI: 49.0–72.0%) for breastfeeding women. When considering the 16 studies that did not distinctly report adherence in these groups, the pooled prevalence of adherence was 75.8% (95% CI: 67.2–82.7%) in pregnant and breastfeeding women in SSA. Examining publication years, there was no discernible difference in prevalence of adherence to option B + ART between studies published after 2018 (73.6%; 95% CI: 69.5–77.3%) and those published before 2018 (67.6%; 95% CI: 55.0–78.0%).

**Table 3:** Subgroup analysis of studies included in the meta-analysis of adherence to option B + ART among pregnant and breastfeeding women in SSA

Subgroup	Number of studies (overall = 42)	Sample size (Total = 15,158)	Pooled adherence % (95%CI)	Heterogeneity		Test for subgroup differences (*p*)
				**I** ^**2**^ ** (%)**	***p***	
**Women’s status**						0.11
Pregnancy	22	7,667	71.9 (66.8–76.4)	94	< 0.01	
Breastfeeding	04	2,299	61.1 (49.0–72.0)	94	< 0.01	
Pregnancy & breastfeeding	16	5,192	75.8 (67.2–82.7)	98	< 0.01	
**Year of publication**						0.32
Before 2018	10	3,927	67.6 (55.0–78.0)	98	< 0.01	
After 2018	32	11,231	73.6 (69.5–77.3)	95	< 0.01	
**Instrument measure**						** < 0.01**
Self-report	26	9,284	75.2 (71.7–78.5)	93	< 0.01	
Combined measures (≥ 2)	07	2,653	75.5 (64.9–83.7)	86	< 0.01	
Pill count/Pharmacy refill	04	763	45.1 (35.3–55.2)	95	< 0.01	
Others**	04	2,328	68.0 (45.9–84.1)	99	< 0.01	
Not reported	01	130^(b)^	-	-	-	
**Regions of SSA**						** < 0.01**
Eastern	17	3,983	74.8 (67.7–80.9)	95	< 0.01	
Southern	14	9,041	70.8 (67.5–73.9)	97	< 0.01	
Western	08	1,720	71.6 (56.9–82.8)	97	< 0.01	
Central	02	315 ^(b)^	-	-	-	
Southern & Western^(a)^	01	99 ^(b)^	-	-	-	
**Design of study**						** < 0.01**
Cross-sectional	29	9,000	76.2 (71.7–80.1)	95	< 0.01	
Cohort	08	3,256	63.0 (54.4–70.9)	94	< 0.01	
RCT	05	2,902	62.8 (47.2–76.1)	98	< 0.01	
**Time frame**						0.23
Last 7 days	16	5,073	75.4 (68.5–81.2)	96	< 0.01	
Last 15–30 days	17	6,530	70.7(62.5–77.7)	98	< 0.01	
Last 90 days	03	2,440	66.5 (61.1–71.6)	80	< 0.01	
Not reported	06	1,115	70.8 (62.8–77.8)	85	< 0.01	
**Quality of study**						**0.01**
Satisfactory	26	11,593	74.2 (68.7–79.0)	98	< 0.01	
Unsatisfactory	14	2,647	69.5 (62.8–75.4)	91	< 0.01	
Good	01	485^(b)^	-	-	-	
Very good	01	170^(b)^	-	-	-	

^(a)^study was conducted in three (03) countries from Southern and Western Africa (Mozambique, South Africa and Mali); ^(b)^Meta-analysis was not performed when the number of studies was ≤ 2;

Bold value means significant subgroup difference;

^**^ others: Dried Blood Spot (DBS); Medication Possession Ratio (MPR); Electronic Medical Records System

Adherence levels significantly varied based on measurement instruments, with notably higher rates (75.5%; 95% CI: 64.9–83.7%) observed when using at least two measurement tools. In contrast, the lowest adherence (45.1%; 95% CI: 35.3–55.2%) was noted when relying on pill count or pharmacy refill measures.

Adherence to option B + ART significantly differed between regions, with the highest pooled prevalence (74.8%; 95% CI: 67.7–80.9%) reported in eastern Africa. Across study designs, the pooled prevalence of adherence was significantly higher in cross-sectional studies (76.2%; 95% CI: 71.7–80.1%), compared to cohort studies (63.0%; 95% CI: 54.4–70.9%), and randomised control trials (62.8%; 95% CI: 47.2–76.1%) studies. Moreover, based on the assessment timeframe, there was no significant difference in the adherence within the last 7 days (75.4%; 68.5–81.2%) and that within the last 90 days (66.5%; 95% CI: 61.1–71.6%).

The multiple meta-regression including all the seven variables indicated that all these variables collectively accounted for 25% of the variation between studies (*R* ^*2*^ = 25.56%; *p* = 0.003).

### Factors associated with adherence to option B + ART

#### Association between adherence and sociodemographic factors

**Fig. 4:**
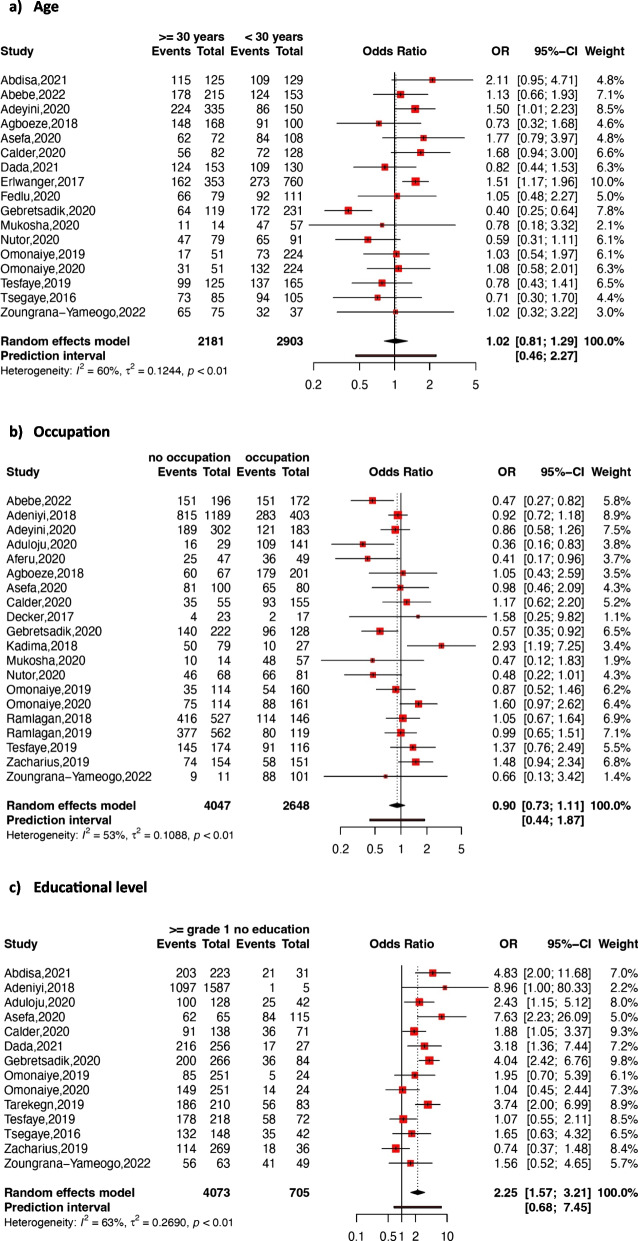
Forest plot of the association between age (**a**), occupation (**b**), educational level (**c**), and adherence to option B + ART

**Fig. 5:**
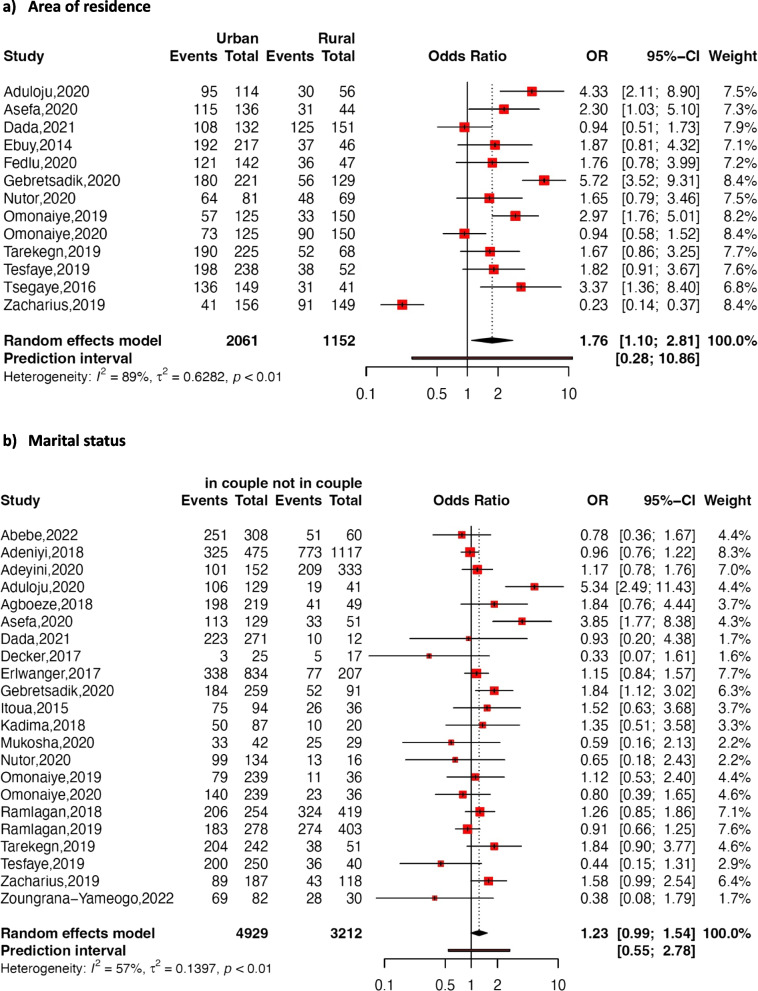
Forest plot of the association between area of residence (**a**), marital status (**b**), and adherence to option B + ART

Figures 4 and 5 present the details of the association between each sociodemographic factor and adherence to option B + ART.

- Age

Seventeen studies were included in the estimation of the pooled association between adherence to option B + ART and women's age. From these studies, only three showed significant associations, with one demonstrating a negative association [47]. Overall, the random-effects pooled association indicated that women’s age was not statistically associated with adherence (pooled OR: 1.02; 95% CI: 0.81–1.29; *p* = 0.84; *I* ^*2*^ = 60%).

- Occupation

Twenty studies were used to determine the pooled association between adherence to option B + ART and women’s occupation. The assessment revealed that women’s occupation was not statistically associated with adherence to option B + ART (pooled OR: 0.90; 95% CI: 0.73*–*1.11; *p* = 0.33; *I* ^*2*^ = 53%).

- Educational level

The random-effects pooled association between adherence to option B + ART and educational status was assessed using fourteen studies. The OR ranged from 0.74 (95% CI: 0.37–1.48) to 8.96 (95% CI: 1.00–80.33). An increased likelihood of adherence was observed among women having at least grade 1 educational level. In fact, those having at least this level of education were significantly more susceptible to adhere compared to those with no educational level background (pooled OR: 2.25; 95% CI: 1.57–3.21; *p* < 0.001; *I* ^*2*^ = 63%).

- Area of residence

The pooled association between adherence to option B + ART and the area of residence was determined using thirteen studies. The random-effects model yielded a pooled OR of 1.76 (95% CI: 1.10–2.81; *p* = 0.019; *I* ^*2*^ = 89%), indicating a higher likelihood of adherence among pregnant or breastfeeding women residing in urban areas compared to those in rural settings.

- Marital status

The pooled association between marital status and adherence to option B + ART was assessed using twenty-two studies, revealing no significant difference in adherence between women in couples (relationships or marriages) and those who were not (pooled OR: 1.23; 95% CI: 0.99–1.53; *p*=0.07; *I* ^*2*^ = 57%).

### Association between adherence to option B + ART and social and clinical factors

**Fig. 6:**
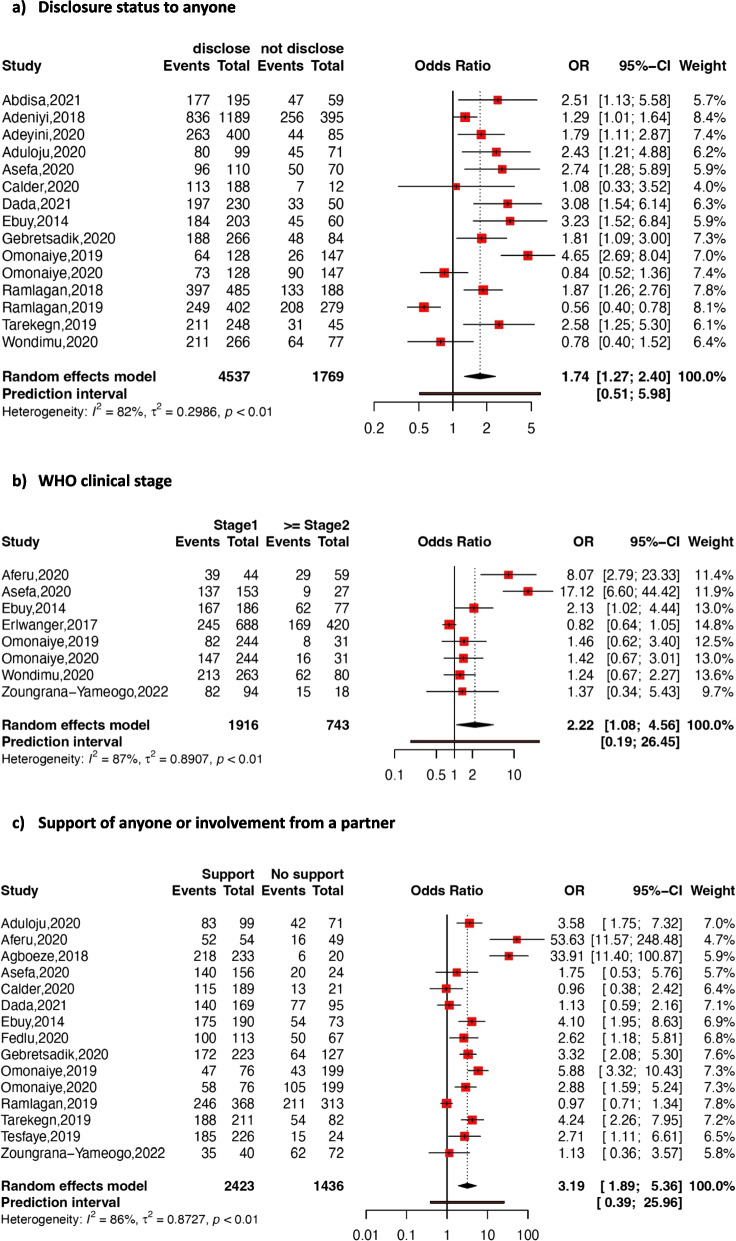
Forest Plot showing association between disclosure status (**a**), WHO clinical stage (**b**), support of anyone or involvement from a partner (**c**), and adherence to option B + ART in pregnant or breastfeeding women in SSA

**Fig. 7:**
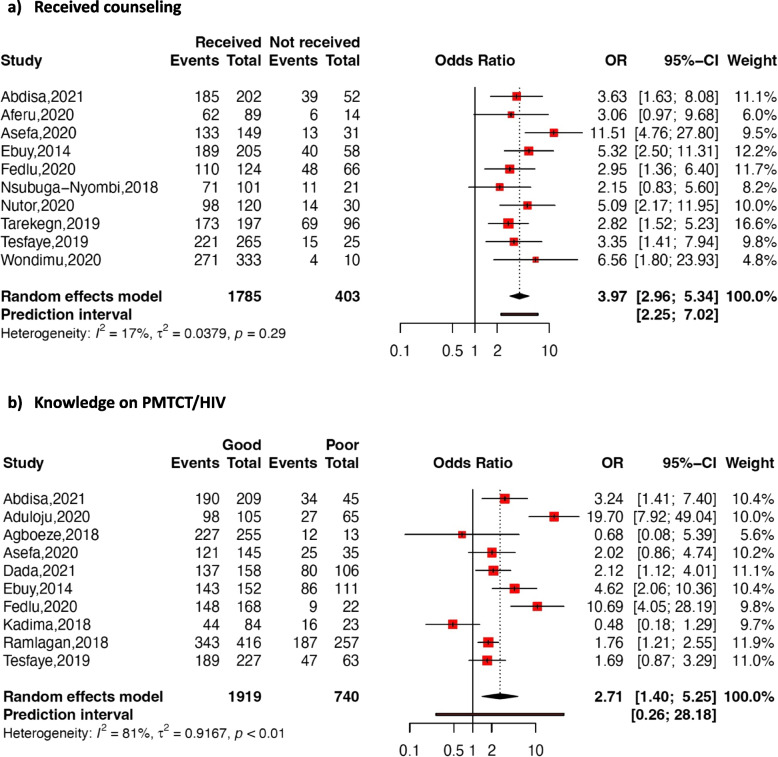
Forest Plot reporting association between receiving counseling (**a**), knowledge on PMTCT/HIV (**b**), and adherence to option B + ART in pregnant or breastfeeding women in SSA

Figures 6 and 7 present details of the association between social/clinical factors and adherence to option B + ART.

- Disclosure status

Fifteen studies were included in the random-effects pooled estimation of the association between adherence to option B + ART and disclosure status. The results revealed that pregnant or breastfeeding women who disclosed their HIV status to a family member or their partner were more likely to adhere to option B + ART compared to those who did not disclose their status (pooled OR: 1.74; 95% CI: 1.27–2.40; *p* < 0.001; *I* ^*2*^ = 82 %).

- Support of anyone or involvement from a partner

The random-effects pooled estimation of the association between adherence to option B + ART and support of anyone or involvement from a partner, including 15 studies revealed a pooled OR of 3.19 (95% CI: 1.89–5.36; *p* < 0.001; *I* ^*2*^ = 86%). This suggests that pregnant, and breastfeeding women receiving support from any person or involvement from a partner are more likely to adhere to option B + ART compared to those lacking such support or involvement.

- Receiving counseling

The pooled association between adherence to option B + ART and receiving counseling was estimated using ten studies. The results showed low heterogeneity among the studies (*I* ^*2*^ = 17 %; *p* = 0.29). The random-effects pooled OR was 3.97 (95% CI: 2.96–5.34; *p* < 0.001), indicating that women who received counseling were more likely to adhere compared to those who did not.

- Clinical stage

Eight studies were included in the estimation of the random-effects pooled association between option B + ART adherence and HIV clinical stage, revealing a pooled OR of 2.22 (95% CI: 1.08–4.56; *p* = 0.03; *I* ^*2*^ = 87%). This suggests that women who initiated treatment at clinical stage 1 were more likely to adhere compared to those initiating treatment at clinical stage 2 or beyond.

- Knowledge on PMTCT/HIV

The random-effects pooled association between option B + ART adherence and knowledge on PMTCT/HIV, involving ten studies revealed a pooled OR of 2.71 (95% CI: 1.40–5.25; p = 0.003; *I* ^*2*^ = 81%), indicating that women with good knowledge on PMTCT/HIV were more likely to adhere to option B + ART compared to those with poor knowledge.

## Discussion

To the best of our knowledge, this is the first study to summarise the available data on adherence to option B + ART in pregnant and breastfeeding women in SSA. The option B + strategy was first implemented in Malawi in 2011, and since then, significant reduction in MTCT has been observed [68]. However, despite the considerable progress observed, adherence to lifelong ART remains a challenge in SSA. In our meta-analysis, the pooled prevalence of adherence to option B + ART among pregnant and breastfeeding women in SSA was estimated to be 72.3% (95% CI: 68.2–76.1%). Tsegaye et al. conducted a meta-analysis in Eastern African countries and reported similar results (71.9%), although their study focused on women in general [69]. Subgroup analyses based on various factors such as women’s status, year of publication, and time frame recall did not show significant differences in option B + ART adherence. However, regional analysis revealed higher adherence in Eastern Africa, potentially influenced by specific interventions designed to improve adherence in that region. Among these interventions, the mother-mentors program stands out. This program involves training HIV-infected mothers who are employed in a healthcare facility to provide one-on-one support to HIV-infected pregnant/postpartum women, encourage enrolment, adherence, and retention in HIV care; leading to easy identification of women missing clinic visits; and educating them on PMTCT and health-related topics [70, 71]. A study conducted in Uganda between 2011 and 2014 revealed that HIV-positive mothers and their HIV-exposed infants enrolled in the mothers2mothers Ugandan Mentor Mother program had higher retention in HIV care at every step of the PMTCT, including adherence to ART [72]. Stratifying by women’s status, our study revealed that pregnant women were more adherent than breastfeeding women. These findings are similar to those of a meta-analysis conducted by Nachega et al. in low-, middle-, and high-income countries, revealing an adherence of 75.7% and 53.0% in pregnant and breastfeeding women, respectively. This difference in the two periods could be due to the fear of women to transmit HIV to their babies through pregnancy. Moreover, women may suffer from postpartum depression (PPD) after pregnancy, negatively affecting adherence to ART [73].

Our meta-analysis has revealed that around one-quarter of pregnant and breastfeeding women in SSA did not have the optimal adherence level needed for successful viral load suppression and, consequently, may be at a higher risk of transmitting HIV to their infants. Thus, evaluating the comprehensive effectiveness of interventions tailored for the prenatal and postpartum periods becomes imperative. Pellowski et al. in their systematic review and meta-analysis published in 2019 revealed limited efficacy of the behavioural ART interventions targeting pregnant and breastfeeding women despite the common motivation associated with childbirth and motherhood [74]. Urgent actions aimed at innovating and reinforcing established interventions could significantly bolster ART adherence in this critical population, especially during the postpartum period marked by insufficient adherence. Our analysis revealed substantial heterogeneity among studies (96.5%). This aligns with similar findings from studies by Nachega et al. in 2012 (97.7%) [29], Tsegaye et al. in 2020 (99%) [69], and de Mattos Costa et al. in 2018 (98%) [75]. This high heterogeneity could be attributed to considerable variations in adherence measurement methods, thresholds, instruments, and definitions employed across the studies.

Several factors associated with adherence to option B + ART in pregnant and breastfeeding women were identified in our meta-analysis. Considering the sociodemographic characteristics, both educational level and area of residence emerged as significantly positively associated with adherence to option B + ART. Pregnant and/or breastfeeding women with higher educational levels were more likely to adhere to option B + ART than those with lower educational levels. Similar results were reported by other studies [14, 16, 23, 60, 76]. However, contrasting trends were reported in some studies [50, 63], despite the lack of statistical significance in these associations. Moreover, pregnant and breastfeeding women living in urban areas exhibited higher adherence compared to those living in rural settings. This result may be due to challenging socioeconomic conditions in rural areas, constraining access to transportation to healthcare facilities [77].

Our meta-analysis revealed that disclosure of HIV status to partner and/or family members were associated with adherence to option B + ART. Pregnant and lactating women disclosing their status to a partner or family member were 1.74 times more likely to adhere to ART than those who do not disclose their status. This positive association has also been reported in other studies. This could be due to the support received after disclosing their status, which encourages continued adherence in the fight against HIV. Additionally, our study highlighted that pregnant and breastfeeding women receiving support or involvement from partners or anyone were three times more likely to adhere to ART than those lacking such support. Similar associations have been reported in previous meta-analyses [69, 75]. However, for this support to be effective, it needs to be consistent and complemented by additional interventions such as structural support or education [70].

Counselling received by pregnant and breastfeeding women emerged as a key predictor of good adherence in our study. This finding aligns with results from a meta-analysis by Wubneh et al. in 2022, indicating that pregnant and breastfeeding women who received counseling during the antenatal period were five times more likely to adhere to ART compared to those who did not receive counseling [78].

Rapid ART initiation is strongly recommended by the WHO, due to its strong association with adherence in adults and adolescents [79]. Clinical stage at ART initiation significantly influences adherence among individuals living with HIV, especially pregnant and breastfeeding women. Our meta-analysis demonstrated that pregnant and breastfeeding women initiating option B + ART at WHO clinical stage I were approximately two times more likely to be adherent compared to those initiating option B + ART at clinical stage II or higher. A study in Northern Ethiopia similarly found that HIV patients starting treatment at stage I were two times (AOR: 2.194; 95% CI: 1.116–4.314) more likely to adhere to prescribed ART compared to those starting at WHO stage IV [80]. This disparity could be linked to an increased medication burden due to additional treatments for possible opportunistic infections that may occur.

A good knowledge on PMTCT or HIV was associated with good adherence in our study. Pregnant and breastfeeding women who had good knowledge on PMTCT and/or HIV were twice as likely to adhere compared to those with limited knowledge. It should be noted that knowledge on PMTCT and/or HIV has been proven to be a predictor of retention in care and, consequently, adherence to ART [81, 82]. The awareness of the benefits of the treatment for them and for their exposed children may increase their willingness to maintain their infants’ safety; pushing to follow their treatment.

Moreover, the results of our moderator analysis revealed that individual-level behaviour plays a substantial role in improving adherence in the pregnancy and postpartum periods. However, a meta-analysis on adherence interventions for women with HIV suggested that in addition to individual-level behaviour, interventions should incorporate structural factors like peer counselor care and additional clinic support staff trained in HIV care to ensure comprehensive follow-up, both at clinics and home [74].

Evidence has shed light on gender-based disparities in the adherence to ART [74, 83]. Women, globally, have been shown to encounter several barriers that impede their adherence to ART, encompassing emotional distress, stigma, negative effects of poor social relationships, and mental health barriers, such as depression, which robustly predicts non-adherence to ART [74, 84]. Furthermore, distinct life events like pregnancy and postpartum periods introduce unique challenges and concerns, making pregnant and/or breastfeeding women more vulnerable to the impact of various barriers on their medication adherence. Our meta-analysis has identified specific barriers within this sub-population, including low educational level, residing in rural areas, non-disclosure of HIV status, lack of social support, inadequate counseling, later initiation of ART, and limited knowledge about HIV and PMTCT. The recognition of these obstacles is vital in designing targeted interventions that address the unique needs of pregnant and breastfeeding women, in order to enhance ART adherence and promote better health outcomes in the Sub-Saharan African context.

### Strengths and limitation of the study

This meta-analysis is the first to determine the pooled proportion of adherence to pregnancy and breastfeeding in the option B + era in SSA. We used various databases to search published studies, and the rigorous methodology of selection facilitated the collection of a large number of articles. Moreover, we strictly followed the PRISMA guidelines for reporting systematic reviews and meta-analyses using a critical appraisal of study quality. Furthermore, no evidence of publication bias was observed, and sensitivity analysis did not reveal a significant change in the prevalence of adherence.

Despite these strengths, our study had some limitations. As we conducted a meta-analysis of observational studies, this study is prone to several biases, including selection and confounding bias in each individual study. Moreover, we observed significant between-study variability (high heterogeneity), indicating significant variations in adherence across studies, probably due to the methodological differences between the studies, including difference in the population, thresholds used, time frames, and measurement methods. To address heterogeneity, we used random-effects models for analysis. Subgroup and meta-regression analyses were used to analyse heterogeneity but did not entirely reveal the source of high heterogeneity. Another limitation is information bias as the main measurement method used in the studies was self-reporting which may have overestimated adherence level and consequently the pooled prevalence of adherence reported in our meta-analysis. Furthermore, we did not include unpublished studies, and therefore, may have missed relevant articles. Lastly, we failed to estimate the prevalence of adherence at different points in the postpartum period since it has been demonstrated that the adherence declines with time.

## Conclusions

In conclusion, our study revealed that three out of ten pregnant and breastfeeding women failed to achieve adequate levels of adherence to option B + ART in SSA. Notably, postpartum period exhibited the lowest adherence rates. To enhance adherence during these pivotal stages, interventions targeting individual factors including educational level, area of residence, disclosure status, WHO clinical stage at ART initiation, support of anyone or involvement from a partner, receiving counseling, and knowledge on PMTCT/HIV are crucial. Additionally, integrating these interventions with structural components, like the presence of peer counselors, is vital for an effective approach.

### Supplementary Information

**Additional file 1.**

## Data Availability

All data used in the analysis are freely and publicly available from the cited papers with full citation listed in the reference section.

## Abbreviations

AACTG: Adult AIDS Clinical Trial Groups
AHRQ: Agency for Healthcare Research and Quality
ART: Antiretroviral Therapy
CASE: Center for Adherence Support Evaluation
DBS: Dry Blood Spot
MMAS: Modified Morisky Medication Adherence Scale
MPR: Medication Possession Ratio
MTCT: Mother-To-Child Transmission
NOS: Newcastle Ottawa Scale
OR: Odds Ratio
PMTCT: Prevention of Mother-To-Child Transmission
SSA: Sub-Saharan Africa
SR: Self-Report
VAS: Visual Analogue Scale
WHO: World Health Organization
