# Supplementary material for: Adherence to option B + antiretroviral therapy and associated factors in pregnant and breastfeeding women in Sub-Saharan Africa: a systematic review and meta-analysis
Source: BMC Public Health. 2024 Jan 5;24:94. doi: 10.1186/s12889-023-17004-9 (PMC10768427; doi:10.1186/s12889-023-17004-9)
Supplement: Supplementary file 1 — Additional file 1. [file 12889_2023_17004_MOESM1_ESM.pdf]

Search strategy in PubMed/Medline:

#1 AND (#2 OR #5) AND #3 AND #4

#1: ("Africa South of the Sahara"[Mesh] OR "Africa South of the Sahara"[Title/Abstract] OR "Subsaharan Africa"[Title/Abstract] OR Cameroon[Title/Abstract] OR "Central African Republic"[Title/Abstract] OR Chad[Title/Abstract] OR Congo[Title/Abstract] OR "Equatorial Guinea"[Title/Abstract] OR Gabon[Title/Abstract] OR "Sao Tome and Principe"[Title/Abstract] OR Burundi[Title/Abstract] OR Djibouti[Title/Abstract] OR Eritrea[Title/Abstract] OR Ethiopia[Title/Abstract] OR Kenya[Title/Abstract] OR Rwanda[Title/Abstract] OR Somalia[Title/Abstract] OR Sudan[Title/Abstract] OR Tanzania[Title/Abstract] OR Uganda[Title/Abstract] OR Angola[Title/Abstract] OR Botswana[Title/Abstract] OR Eswatini[Title/Abstract] OR Lesotho[Title/Abstract] OR Malawi[Title/Abstract] OR Mozambique[Title/Abstract] OR Namibia[Title/Abstract] OR "South Africa"[Title/Abstract] OR Zambia[Title/Abstract] OR Zimbabwe[Title/Abstract] OR Benin[Title/Abstract] OR "Burkina Faso"[Title/Abstract] OR "Cabo Verde"[Title/Abstract] OR "Cote d'Ivoire"[Title/Abstract] OR Gambia[Title/Abstract] OR Ghana[Title/Abstract] OR Guinea[Title/Abstract] OR Liberia[Title/Abstract] OR Mali[Title/Abstract] OR Mauritania[Title/Abstract] OR Niger[Title/Abstract] OR Nigeria[Title/Abstract] OR Senegal[Title/Abstract] OR "Sierra Leone"[Title/Abstract] OR Togo[Title/Abstract] OR "East Africa"[Title/Abstract] OR "Eastern Africa"[Title/Abstract] OR "Eastern African"[Title/Abstract])

#2: ("Infectious Disease Transmission, Vertical/prevention and control"[Mesh] OR PMTCT[Title/Abstract] OR (("Vertical Transmission of Infectious Disease"[Title/Abstract] OR "Vertical Infection Transmission"[Title/Abstract] OR "Vertical Infectious Disease Transmission"[Title/Abstract] OR "Maternal Fetal Infection Transmission"[Title/Abstract] OR "Mother to Child Transmission"[Title/Abstract] OR "Fetomaternal Infection Transmission"[Title/Abstract]) AND preventi*[Title/Abstract]))

#3: "Anti Retroviral Agents"[Title/Abstract] OR "Antiretroviral Agents"[Title/Abstract] OR "Highly Active Antiretroviral Therapy"[Title/Abstract] OR "HAART"[Title/Abstract] OR "Combination Antiretroviral Therapy"[Title/Abstract] OR "Combination Antiretroviral Therapies"[Title/Abstract] OR "Anti-Retroviral Agents"[Mesh] OR "Antiretroviral Therapy, Highly Active"[Mesh] OR Antiretroviral* [Title/Abstract] OR ARV[Title/Abstract] OR ART[Title/Abstract] OR "Anti-HIV Agents"[Mesh] OR "Anti HIV Agent"[Title/Abstract] OR "Anti HIV Agent"[Mesh] OR "AIDS Drug"[Title/Abstract] OR "Anti AIDS Drug "[Title/Abstract] OR "Anti-AIDS Agents"[Title/Abstract] OR "Anti-HIV Drug "[Title/Abstract] OR "option B"[Title/Abstract]

#4: "adherence to medications"[Title/Abstract] OR "Drug Adherence "[Title/Abstract] OR "Medication adherence"[Title/Abstract] OR " Medication Nonadherence"[Title/Abstract] OR "Medication Noncompliance"[Title/Abstract] OR "Medication Non-Adherence"[Title/Abstract] OR "Medication Persistence"[Title/Abstract] OR "Medication Compliance"[Title/Abstract] OR "Medication Non-Compliance"[Title/Abstract] OR "Drug Compliance"[Title/Abstract] OR "Patient Adherence"[Title/Abstract] OR "Patient Cooperation"[Title/Abstract] OR "Client Compliance"[Title/Abstract] OR "Client Adherence"[Title/Abstract] OR "Treatment

Compliance"[Title/Abstract] OR "Therapeutic Compliance"[Title/Abstract] OR "Therapeutic Compliances"[Title/Abstract] OR "Patient Non Compliance"[Title/Abstract] OR "Patient NonCompliance"[Title/Abstract] OR "Patient Nonadherence"[Title/Abstract] OR "Non Adherent Patient"[Title/Abstract] OR "Patient Non Adherence"[Title/Abstract] OR "adherence to antiretroviral therapy "[Title/Abstract] OR "ART adherence"[Title/Abstract] OR "medication adherence"[Mesh] OR "Patient Compliance"[Mesh]

#5: (Pregnancy[Mesh] OR "Pregnant Women"[Mesh] OR Pregnan*[Title/Abstract] OR " Perinatal Care"[Mesh] OR perinatal*[Title/Abstract] OR "Breast Feeding"[Mesh] OR Breastfe*[Title/Abstract] OR " Breast Fed"[Title/Abstract] OR " Wet Nursing"[Title/Abstract] OR "Postpartum period"[Mesh] OR Postpartum*[Title/Abstract] OR "Puerperium"[Title/Abstract])

Search strategy in Scopus:

#1 AND (#2 OR #5) AND #3 AND #4

#1: TITLE-ABS-KEY ("Africa South of the Sahara" OR "Subsaharan Africa" OR Cameroon OR "Central African Republic" OR Chad OR Congo OR "Equatorial Guinea" OR Gabon OR "Sao Tome and Principe" OR Burundi OR Djibouti OR Eritrea OR Ethiopia OR Kenya OR Rwanda OR Somalia OR Sudan OR Tanzania OR Uganda OR Angola OR Botswana OR Eswatini OR Lesotho OR Malawi OR Mozambique OR Namibia OR "South Africa" OR Zambia OR Zimbabwe OR Benin OR "Burkina Faso" OR "Cabo Verde" OR "Cote d’Ivoire" OR Gambia OR Ghana OR Guinea OR Liberia OR Mali OR Mauritania OR Niger OR Nigeria OR Senegal OR "Sierra Leone" OR Togo OR "East Africa" OR "Eastern Africa" OR "Eastern African")

#2: TITLE-ABS-KEY (PMTCT OR (("Vertical Transmission of Infectious Disease" OR "Vertical Infection Transmission" OR "Vertical Infectious Disease Transmission" OR "Maternal Fetal Infection Transmission" OR "Mother to Child Transmission" OR "Fetomaternal Infection Transmission") AND preventi*))

#3: TITLE-ABS-KEY ("Anti Retroviral Agents" OR "Antiretroviral Agents" OR "Highly Active Antiretroviral Therapy" OR "HAART" OR "Combination Antiretroviral Therapy" OR "Combination Antiretroviral Therapies" OR Antiretroviral* OR ARV OR ART OR "Anti HIV Agent" OR "AIDS Drug" OR "Anti AIDS Drug " OR "Anti-AIDS Agents" OR "Anti-HIV Drug " OR "option B")

#4: TITLE-ABS-KEY ("adherence to medications" OR "Drug Adherence " OR "Medication adherence" OR "Medication Nonadherence" OR "Medication Noncompliance" OR "Medication Non-Adherence" OR "Medication Persistence" OR "Medication Compliance" OR "Medication Non-Compliance" OR "Drug Compliance" OR "Patient Adherence" OR "Patient Cooperation" OR "Client Compliance" OR "Client Adherence" OR "Treatment Compliance" OR "Therapeutic Compliance" OR "Therapeutic Compliances" OR "Patient Non Compliance" OR "Patient NonCompliance" OR "Patient Nonadherence" OR "Non Adherent Patient" OR "Patient Non Adherence" OR "adherence to antiretroviral therapy " OR "ART adherence")

#5: TITLE-ABS-KEY (Pregnan* OR perinatal* OR Breastfe* OR "Breast Fed" OR " Wet Nursing" OR Postpartum* OR "Puerperium")

Search strategy in proquest central

PMTCT OR (preventi* AND("Vertical Transmission of Infectious Disease" OR "Vertical Infection Transmission" OR "Vertical Infectious Disease Transmission" OR "Maternal Fetal Infection Transmission" OR "Mother to Child Transmission" OR "Fetomaternal Infection Transmission")) OR (Pregnan* OR perinatal* OR Breastfe* OR "Breast Fed" OR "Wet Nursing" OR Postpartum* OR "Puerperium")

AND

"Anti Retroviral Agents" OR "Antiretroviral Agents" OR "Highly Active Antiretroviral Therapy" OR "HAART" OR "Combination Antiretroviral Therapy" OR "Combination Antiretroviral Therapies" OR Antiretroviral* OR ARV OR ART OR "Anti HIV Agent" OR "AIDS Drug" OR "Anti AIDS Drug " OR "Anti-AIDS Agents" OR "Anti-HIV Drug " OR "option B"

AND

"adherence to medications" OR "Drug Adherence " OR "Medication adherence" OR " Medication Nonadherence" OR "Medication Noncompliance" OR "Medication Non-Adherence" OR "Medication Persistence" OR "Medication Compliance" OR "Medication Non-Compliance" OR "Drug Compliance" OR "Patient Adherence" OR "Patient Cooperation" OR "Client Compliance" OR "Client Adherence" OR "Treatment Compliance" OR "Therapeutic Compliance" OR "Therapeutic Compliances" OR "Patient Non Compliance" OR "Patient NonCompliance" OR "Patient Nonadherence" OR "Non Adherent Patient" OR "Patient Non Adherence" OR "adherence to antiretroviral therapy " OR "ART adherence" OR Adherence OR compliance OR persistence OR "non adherence" OR "non compliance" OR "non persistence"

AND

"Africa South of the Sahara" OR "Subsaharan Africa" OR Cameroon OR "Central African Republic" OR Chad OR Congo OR "Equatorial Guinea" OR Gabon OR "Sao Tome and Principe" OR Burundi OR Djibouti OR Eritrea OR Ethiopia OR Kenya OR Rwanda OR Somalia OR Sudan OR Tanzania OR Uganda OR Angola OR Botswana OR Eswatini OR Lesotho OR Malawi OR Mozambique OR Namibia OR "South Africa" OR Zambia OR Zimbabwe OR Benin OR "Burkina Faso" OR "Cabo Verde" OR "Cote d'Ivoire" OR Gambia OR Ghana OR Guinea OR Liberia OR Mali OR Mauritania OR Niger OR Nigeria OR Senegal OR "Sierra Leone" OR Togo OR "East Africa" OR "Eastern Africa" OR "Eastern African"

Search strategy in Index Medicus Africain (IMA)

(PMTCT OR (preventi* AND("Vertical Transmission of Infectious Disease" OR "Vertical Infection Transmission" OR "Vertical Infectious Disease Transmission" OR "Maternal Fetal Infection Transmission" OR "Mother to Child Transmission" OR "Fetomaternal Infection Transmission")) OR (Pregnan* OR perinatal* OR Breastfe* OR "Breast Fed" OR "Wet Nursing" OR Postpartum* OR "Puerperium"))

AND

("Anti Retroviral Agents" OR "Antiretroviral Agents" OR "Highly Active Antiretroviral Therapy" OR "HAART" OR "Combination Antiretroviral Therapy" OR "Combination Antiretroviral Therapies" OR Antiretroviral* OR ARV OR ART OR "Anti HIV Agent" OR "AIDS Drug" OR "Anti AIDS Drug " OR "Anti-AIDS Agents" OR "Anti-HIV Drug " OR "option B")

AND

("adherence to medications" OR "Drug Adherence " OR "Medication adherence" OR " Medication Nonadherence" OR "Medication Noncompliance" OR "Medication Non-Adherence" OR "Medication Persistence" OR "Medication Compliance" OR "Medication Non-Compliance" OR "Drug Compliance" OR "Patient Adherence" OR "Patient Cooperation" OR "Client Compliance" OR "Client Adherence" OR "Treatment Compliance" OR "Therapeutic Compliance" OR "Therapeutic Compliances" OR "Patient Non Compliance" OR "Patient NonCompliance" OR "Patient Nonadherence" OR "Non Adherent Patient" OR "Patient Non Adherence" OR "adherence to antiretroviral therapy " OR "ART adherence" OR Adherence OR compliance OR persistence OR "non adherence" OR "non compliance" OR "non persistence")

Results of the search for each database

**Database**	**Number of articles found**
Pubmed	355
Scopus	346
Proquest Central	901
Index Medicus Africain	09
**Total**	**1611**
